# Progress of the Temperance Reform

**Published:** 1840-10

**Authors:** 


### PROGRESS OF THE TEMPERANCE REFORM.

Within the last six months, we have had ample opportunities of observing the present state of the temperance reform in the Valley of the Ohio—emphatically the heart of the West. Everywhere the friends of temperance appear to be languid, and, in most places, absolutely inert. This does not strike us as remarkable; for nothing is more difficult than to sustain an associated benevolent effort for a great length of time. All such efforts are essentially intermittent. We look for a revival at no distant day. Some new influence will arise, and reproduce the noble warfare of the past.—Meanwhile, we are enabled to say, that in almost every part of the West, that warfare, has been most effective. A careful inquiry of physicians, and intelligent observers of all classes, has convinced us, that the amount of drinking is far less than it was twelve years ago, when the first combinations against it were organized among us. Few or no families now take their morning bitters and, yet, the ague and fever is rather on the decrease than the increase; the practice of offering intoxicating drinks to visiters, has signally declined; the amount drunk at the tables of our taverns and steamboats, has diminished in an equal degree; labouring men take far less than they formerly did—a great number of farmers even give none in harvest time, and still find their operatives much more efficient and enduring, than under the old regimen; at our Fourth of July celebrations, but little is drunk compared with what our fathers consumed on that national festival; the great political meetings of both parties have been characterized by temperance in drinking, whatever may have been the intemperance of their partisan feelings; finally a "drunken doctor" is becoming a phenomenon, which the boys are beginning to chase in the street!

Such are the fruits of a few years exertion, and we conscientiously believe them as rich and abundant as the most sanguine friends of reform ever anticipated. We must not forget, however, that the disease remains uneradicated. The inflammation has been subdued into a subacute and chronic form; but such a morbid action may do the work of destruction, and is at all times liable to be awakened into activity. We call upon our brethren, especially, to be vigilant and tireless in this labour of love and duty. Of all the professions, theirs is the one which may exert the greatest corrective power, and *therefore* lies under the heaviest responsibility. D.

